# A rare case report of an endobronchial teratoma of the right upper lobe invading the mediastinum

**DOI:** 10.1097/RC9.0000000000000851

**Published:** 2026-08-24

**Authors:** Olalekan Babalola, Nathan Siewert, Max Frenkel, Tim Guenther, Malcolm M. DeCamp

**Affiliations:** a University of Wisconsin School of Medicine and Public Health, Madison, WI, USA; b Division of Cardiothoracic Surgery Department of Surgery, University of Wisconsin School of Medicine and Public Health, Madison, WI, USA

**Keywords:** case report, endobronchial teratoma (EBT), intrapulmonary teratoma (IPT), mature teratoma

## Abstract

**Introduction:**

Endobronchial teratomas are extremely rare tumors that develop within the airway of the lung. Patients often present with nonspecific symptoms that depend on the size and anatomic features, making the differential broad and the diagnosis challenging.

**Presentation of case:**

A 28-year-old man presented with a 6-month history of a dry cough, vague chest discomfort, and occasional dyspnea. A chest X-ray and CT were performed, revealing a heterogeneous mass involving the mediastinum and the medial aspect of the right lung. Serum tumor markers were normal, and a testicular ultrasound excluded a metastatic germ cell tumor. Definitive treatment required an *en bloc* resection of the thymus and right upper lobe, with pathologic review showing an endobronchial origin of a mature teratoma.

**Discussion:**

This case illustrates the diagnostic challenges of endobronchial teratomas, provides imaging and histologic characteristics of this rare tumor type, and contributes to the number of published cases with favorable outcomes.

**Conclusion:**

Given the rarity of endobronchial teratomas, a high index of suspicion among pulmonologists, surgeons, and pathologists is necessary to correctly diagnose and treat these patients.

## Introduction

Teratomas are tumors that contain all three germinal layers, and most arise from the ovary, testis, sacrococcygeal region, or mediastinum[1]. Intrapulmonary teratomas are much less common, and fewer than 100 case reports have been published[2]. These can present with nonspecific symptoms such as cough, chest pain, sputum production, and fever. Even more uncommon are teratomas that arise from the airways, known as endobronchial teratomas. These extremely rare tumors can cause not only similar symptoms to intrapulmonary teratomas but also symptoms such as post-obstructive pneumonia, hemoptysis, and trichoptysis (coughing up hair)[3]. We present a case of a suspicious mediastinal mass with suspected local invasion into the lung, airway, and mediastinum that was completely resected with an *en blo*c radical thymectomy, right upper lobectomy, and partial pericardiectomy, which, on pathologic assessment, was found to be a benign endobronchial teratoma. This case was reported in line with the SCARE criteria[4].

HIGHLIGHTS

- Teratomas are tumors that contain all three germinal layers, and most arise from the ovary, testis, sacrococcygeal region, or mediastinum.
- Teratomas that arise from the airways are known as endobronchial teratomas. These extremely rare tumors cause symptoms such as post-obstructive pneumonia, hemoptysis, and trichoptysis (coughing up hair).
- Surgical resection remains the gold-standard treatment for endobronchial teratomas, given the mass effect, the risk of rupture into the bronchial tree or pleural space, and the potential for malignant transformation.

## Presentation of case

**Figure 1.**
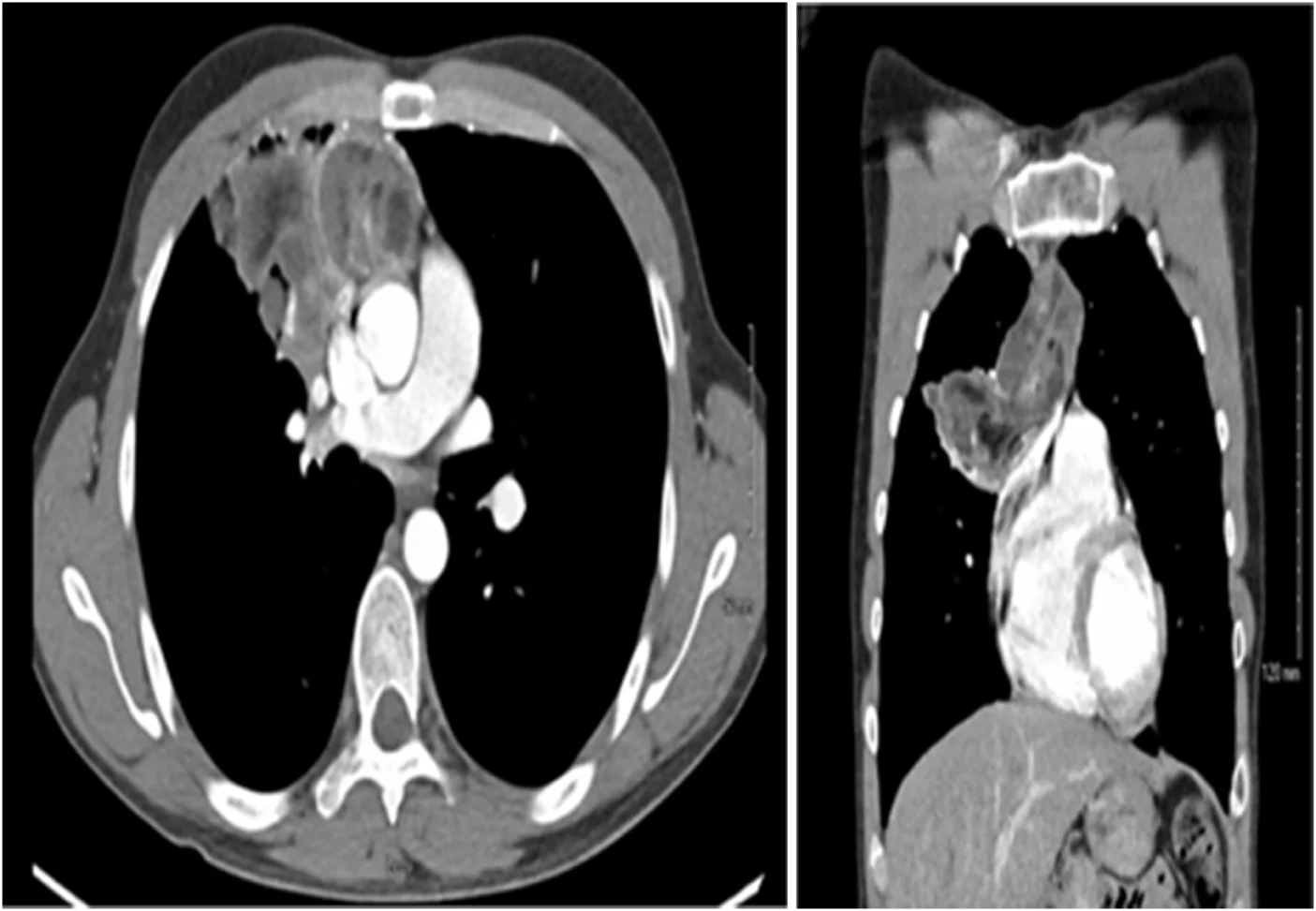
Preoperative chest CT scan showing a 7.9 × 5.8 × 9.7 cm heterogeneous anterior mediastinal mass containing fat, soft-tissue elements, blood vessels, and calcification in axial and coronal sections.

A 28-year-old man was referred to our clinic after an emergency room visit for chest pain, during which a chest X-ray and a contrast-enhanced computed tomography (CT) scan were performed. He reported a 6-month history of a non-productive cough and reduced exercise tolerance. The patient had a brief history of tobacco use and had quit approximately 10 years earlier. Past medical, surgical, and family histories were unremarkable. Physical examination was unremarkable. Laboratory tests, including alpha-fetoprotein and beta-human chorionic gonadotropin levels, were normal. A contrast-enhanced CT scan showed a 7.9 × 5.8 × 9.7 cm heterogeneous anterior mediastinal mass containing fat, soft tissue elements, blood vessels, and calcifications. The mass extended inferolaterally from the thymus at the level of the left brachiocephalic vein and invaded the right upper lobe (Fig. 1). These features were suspicious for malignancy. A testicular examination and ultrasound were normal, excluding a metastatic germ cell tumor. After a multidisciplinary tumor board discussion, the decision was made to proceed with resection given the size of the mass and the patient’s symptoms.

**Figure 2.**
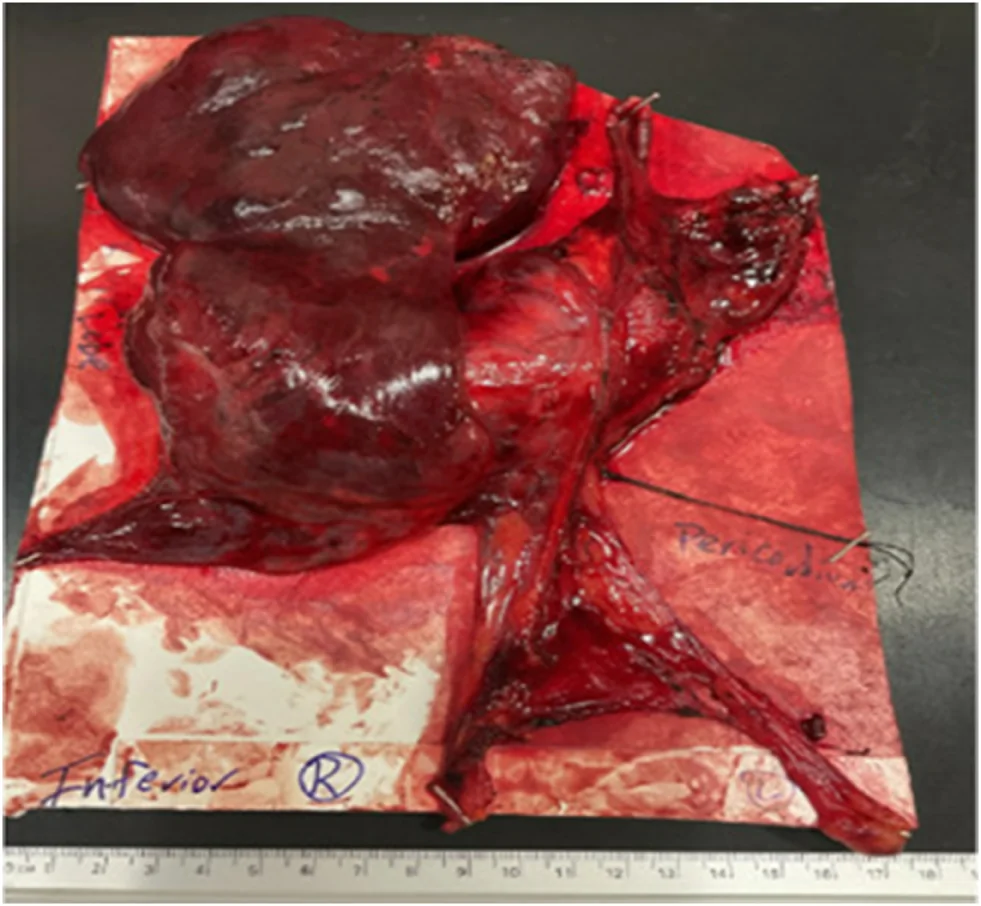
Resected right upper lobe, with thymus and pericardium harvested *en bloc*.

Pre-operative bronchoscopy was unremarkable to the level of the bilateral segmental bronchi. The mass was approached via a right hemi-clamshell sterno-thoracotomy and appeared to involve the anterior mediastinum with direct extension into the right upper lobe. To completely remove the mass, an *en bloc* right upper lobectomy and radical thymectomy were performed. Because of dense adhesions, an 8 × 10-cm piece of pericardium was included with the resected mass; however, the right phrenic nerve was spared (Fig. 2).

**Figure 3.**
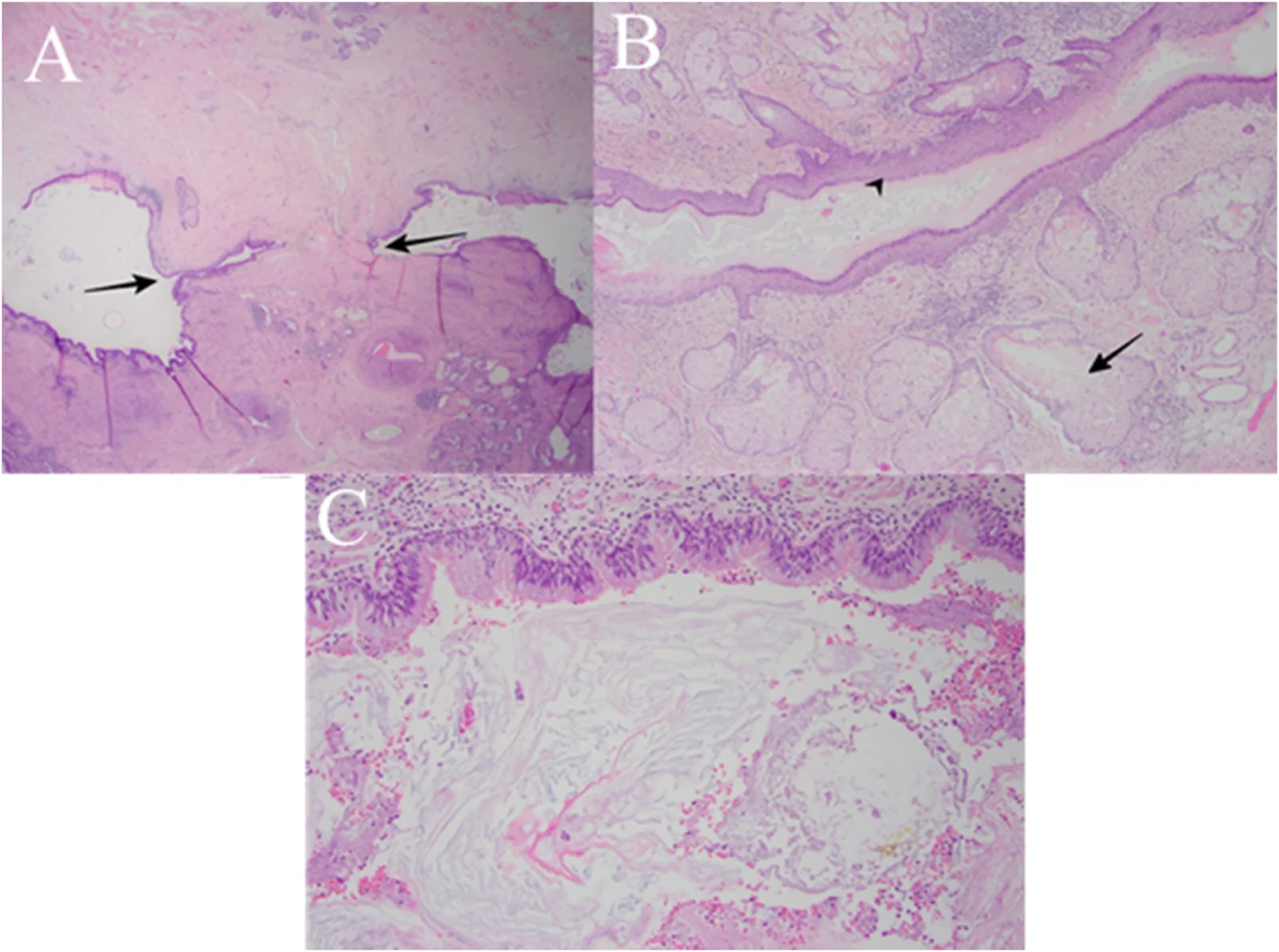
Hematoxylin- and eosin-stained sections demonstrating endobronchial teratoma with characteristic cellular features: (A) teratoma (top of the image) connected to the airway (bottom of the image) by a narrow stalk (arrows), 12.5× magnification; (B) teratoma with features of mature skin (arrowhead) and sebaceous glands (arrow), 100× magnification; (C) endobronchial keratinous debris, 200× magnification.

All margins were inked prior to tissue fixation, and an extensive histopathologic review was performed, confirming a benign mature teratoma with microscopically negative margins and no lymph node involvement. The teratoma appeared to arise within an airway of the right upper lobe, with direct extension into mediastinal tissue, including the thymus (Fig. 3). The adjacent lung parenchyma showed post-obstructive changes and exogenous lipoid pneumonia.

The patient recovered without complications and was discharged on postoperative day three. The patient was discussed again in a multi-disciplinary setting, and because all teratomatous elements were mature and the resection was complete, adjuvant therapy was not pursued. At his 4-month follow-up visit, the patient is doing well, with well-healed wounds, no pain, resolution of his cough, and he is fully active.

## Discussion

Intrapulmonary teratomas are thought to arise from aberrant migration of cells from the third pharyngeal pouch during embryogenesis, which may explain the commonly observed thymic component[5]. This same aberrant migration near the tracheobronchial tree likely leads to the formation of endobronchial teratomas. Endobronchial teratomas most commonly involve the left upper lobe bronchus, but they can theoretically arise anywhere along the tracheobronchial tree[6].

Intrapulmonary and endobronchial teratomas can be difficult to identify at initial presentation because they clinically and radiographically overlap with many other thoracic conditions. Chest CT is particularly valuable for diagnosis, as it can identify characteristic features for teratomas, such as the combination of fat, soft tissue, fluid, calcification, teeth, and bone. However, a definitive diagnosis still requires pathologic evaluation, since intrapulmonary teratomas can mimic other intrapulmonary tumors, including thymomas, primary lung cancers, and metastases[7]. Pre-operative biopsy may or may not be necessary depending on the clinical presentation. This step was not pursued in our patient because the tumor markers were negative and it was felt that a biopsy would not change management, as resection would be necessary given the size of the mass and the patient’s symptoms.

Surgical resection remains the gold standard treatment for intrapulmonary and endobronchial teratomas given the mass effect, the risk of rupture into the bronchial tree or pleural space, and the potential for malignant transformation[8]. Given their rarity, a pre-operative diagnosis of these tumors may not be possible and is only obtained after final pathologic assessment. Adjuvant therapy following completely resected benign intra-pulmonary or endobronchial teratomas is generally not pursued[9]. Fortunately, the prognosis following resection of mature intrapulmonary and endobronchial teratomas is generally favorable, with a low likelihood of malignancy or recurrence following complete resection.

## Conclusion

Our case presents a unique case of an endobronchial teratoma involving the right upper lobe that was diagnosed on final pathology after *en bloc* resection of the thymus, right upper lobe, and pericardium. This case adds to the literature on this rare disease and highlights the utility of high-resolution imaging for pre-operative planning and careful histopathologic review to define the true origin of a mature teratoma.

## Data Availability

Not applicable.
